# Holocaine

**Published:** 1899-05

**Authors:** 


Holocaine. This is a new local anæsthetic, and is offered as a substitute for cocaine. It occurs in the form of white needle-like crystals which are sparingly soluble in cold water, very readily soluble in hot water and alcohol. For ophthalmic practice a 1 per cent. solution is recommended, two or three drops of which produce anæsthesia in from forty to fifty seconds. The anæsthetic effect lasts about five minutes.

